# A Challenging Case of Solitary Necrotic Nodules of the Liver Mimicking Hepatic Metastases: CT, MRI, and PET-CT Findings

**DOI:** 10.5334/jbsr.2088

**Published:** 2020-04-07

**Authors:** Ji Yeon Hwang, Ji Eun Lee, Min Jung Jung

**Affiliations:** 1 Soonchunhyang University College of Medicine, Bucheon Hospital, Bucheon, KR

**Keywords:** solitary necrotic nodule, liver metastasis, computed tomography, magnetic resonance imaging, positron-emission tomography/computed tomography

## Abstract

**Main teaching point**: Solitary necrotic nodules of the liver can be multiple and usually appear as hypovascular nodules mimicking hepatic metastases, but they are relatively small and located in the subcapsular areas of the liver.

We report a very rare case of multiple solitary necrotic nodules of the liver mimicking hepatic metastases in a patient with previous history of lung cancer. The lesions appeared as low-attenuated or low-signal intensity nodules with thin rim enhancement on both contrast-enhanced computed tomography (CT) and magnetic resonance (MR) imaging, making them difficult to differentiate from hepatic metastases. This rare benign entity should be kept in mind, especially when lesions are small and located in the subcapsular areas of the liver.

## Introduction

Solitary necrotic nodule of the liver is an extremely rare lesion that is benign but commonly mistaken as malignancy, such as hepatic metastases, due to its nonspecific imaging findings. First reported by Shepherd et al. in 1983 [1], solitary necrotic nodule is characterized as a lesion with a completely necrotic core, is encapsulated by fibrotic tissue, and contains collagen, elastic fibers, and inflammatory cells. The etiology remains uncertain, but various hypotheses, such as infection, trauma, or regression from sclerosing hemangioma, have been suggested [2,3]. A solitary necrotic nodule has no characteristic clinical or radiological features, so it is difficult for clinicians to achieve an accurate preoperative diagnosis and differentiate the lesion from hepatic metastases, especially when the patient has previous history of malignancy or multiple lesions. Thus, we present a very challenging case of a 64-year-old woman with past medical history of lung cancer who had two solitary necrotic nodules of the liver that were surgically resected due to imaging findings that mimicked hepatic metastases.

## Case report

A 64-year-old woman was referred to our hospital for incidentally discovered hypoechoic hepatic nodules on abdominal ultrasound (not shown). Her past medical history was significant for surgery and chemotherapy for adenocarcinoma of the lung 12 years prior. The patient had no clinical symptoms at presentation. Laboratory tests showed elevated serum carbohydrate antigen 19–9 (CA 19–9) of 42.2 U/mL (normal range: 0–27 U/mL). All other laboratory tests, including complete blood count (CBC), electrolytes, aspartate aminotransferase (AST), and alanine aminotransferase (ALT), were within normal limits. The hepatitis virus serological tests were also normal.

**Figure 1:**
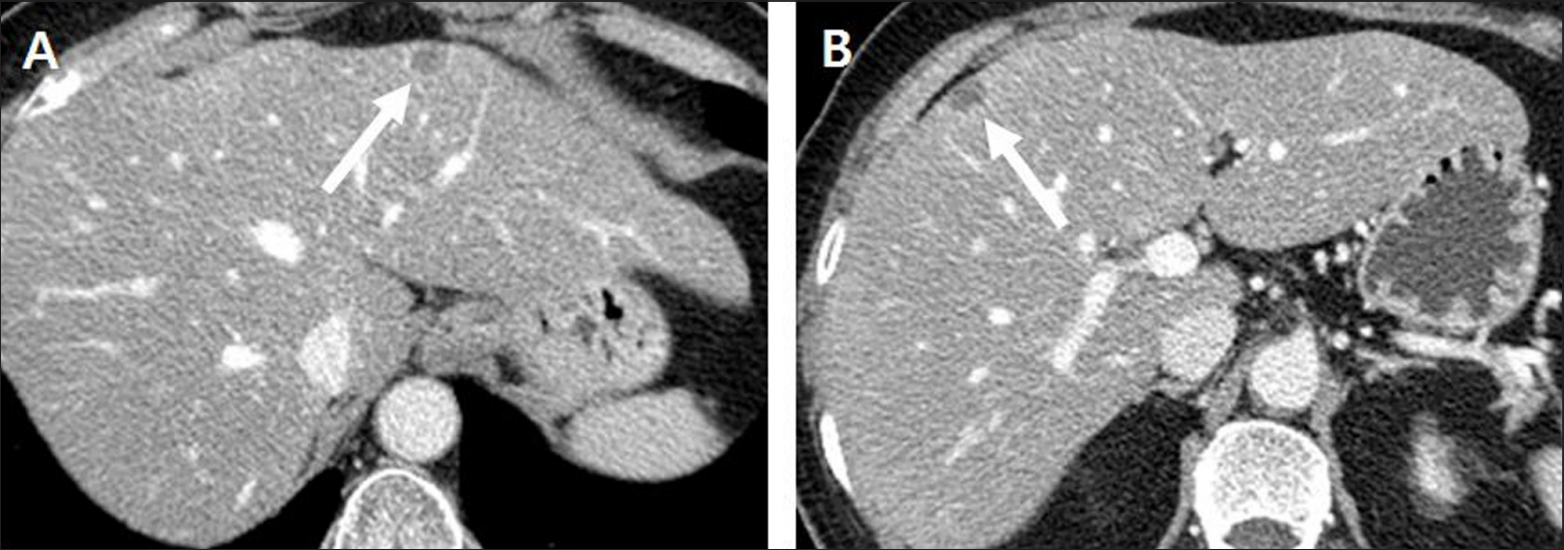
Contrast-enhanced axial CT (computed tomography) images in the portal phase **(A, B)** showed two small, rim-enhancing, low-attenuated nodules *(arrow)* in the subcapsular area of liver segments 2 and 8.

**Figure 2:**
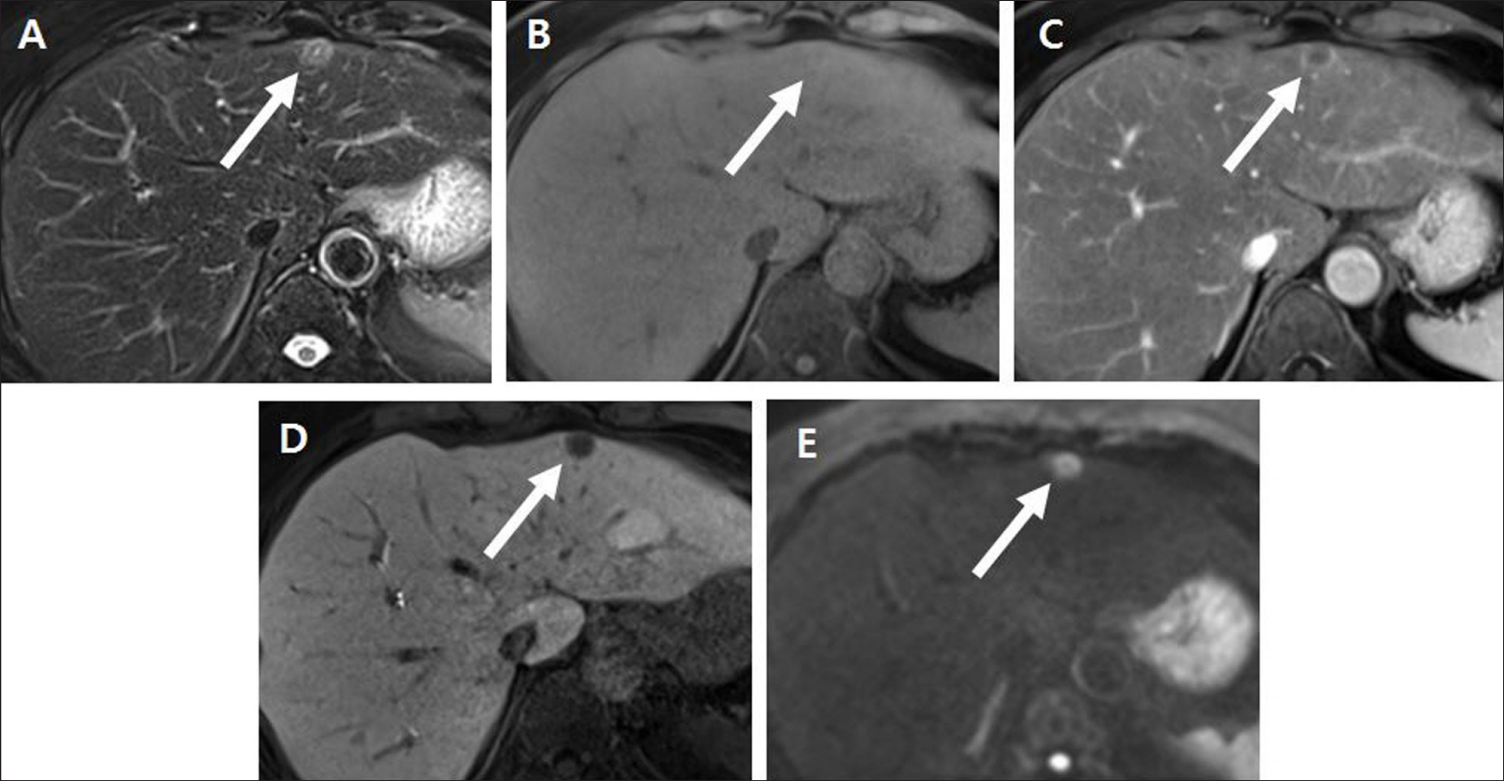
**(A)** Axial, respiratory-triggered, single-shot, T2-weighted MR (magnetic resonance) imaging revealed a nodule *(arrow)* in liver segment 2, showing a target appearance with intermediate high signal intensity and central, dot-like, bright signal intensity. **(B)** On unenhanced, T1-weighted MR imaging, the lesion *(arrow)* was nearly iso-signal intensity to the normal liver parenchyma. **(C)** On contrast-enhanced arterial phase MR imaging, the lesion *(arrow)* showed rim-enhancement. **(D)** On the hepatobiliary phase, the lesion *(arrow)* demonstrated low signal intensity. **(E)** On diffusion-weighted image (b = 800 s/mm^2^), the lesion *(arrow)* showed high signal intensity.

**Figure 3:**
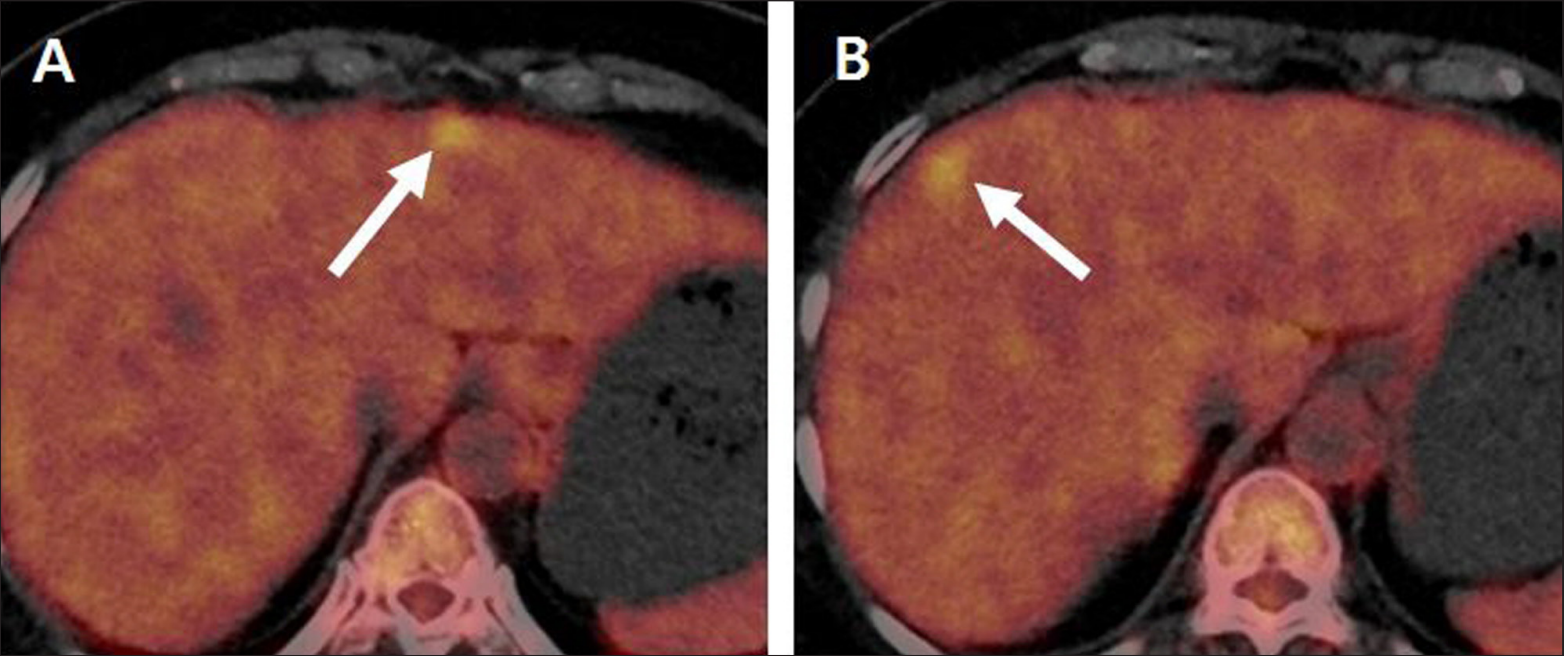
On 2-[^18^F]-fluoro-2-deoxy-D-glucose (FDG) positron-emission tomography (PET)/CT **(A, B)**, both lesions showed increased FDG uptake.

For further evaluation, abdominal computed tomography (CT) and magnetic resonance (MR) imaging were performed. Contrast-enhanced CT revealed two, approximately 1.2-cm-sized, low-attenuated nodules showing rim-enhancement in liver segments 2 and 8 (Figure 1). On MR imaging, both nodules showed intermediate high signal intensity on T2-weighted images (T2WI), and the lesion in segment 2 showed central cystic change with T2 bright signal intensity (Figure 2A). Both nodules showed iso-signal intensity on T1WI (Figure 2B) with rim-enhancement on arterial phase of gadoxetic acid-enhanced MR imaging (Figure 2C) and low signal intensity on hepatobiliary phase imaging (Figure 2D). On diffusion-weighted imaging with a b-value of 800 s/mm^2^, the lesions showed high signal intensity (Figure 2E) and 2-[^18^F]-fluoro-2-deoxy-D-glucose (FDG) positron-emission tomography (PET)/CT revealed mild hypermetabolism of the lesions (Figure 3). Therefore, both lesions were interpreted as hepatic metastases and surgically resected.

**Figure 4:**
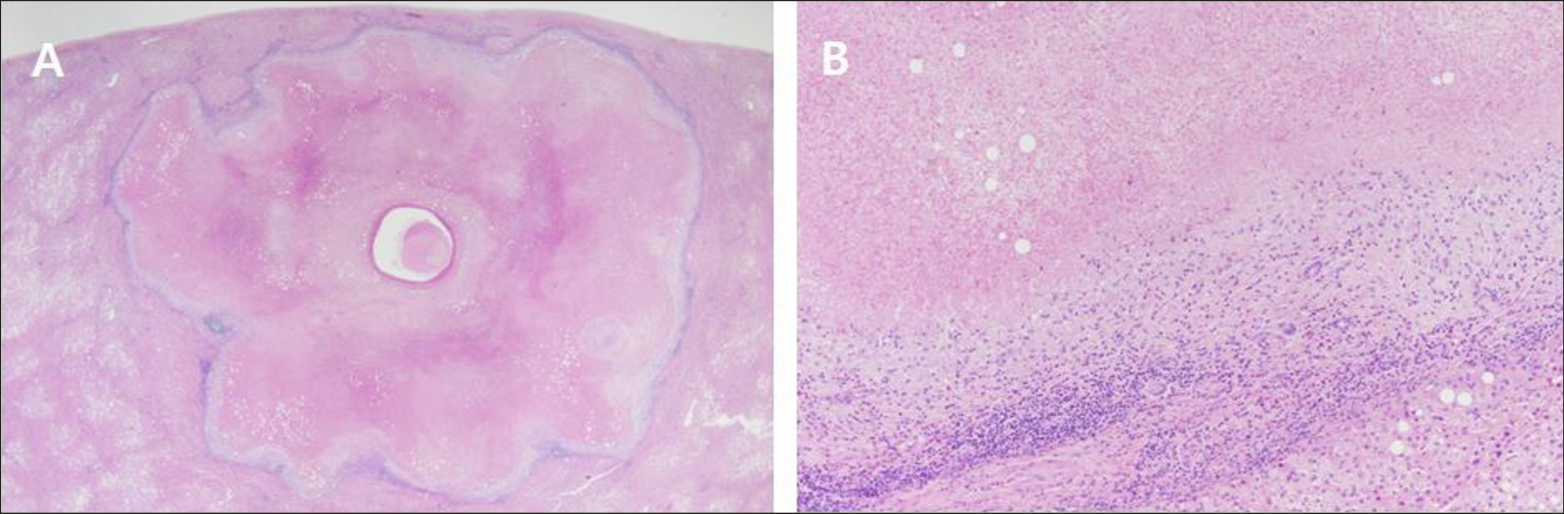
**(A)** Scan power view of the liver specimen revealed a necrotic nodule with central cystic space and surrounded by fibrous tissue (Hematoxylin and eosin, ×10). **(B)** The edge of the nodule showed granulomatous inflammation consisting of epithelioid histiocytes, lymphocytes, some eosinophils, and a few giant cells (Hematoxylin and eosin, ×100).

The patient underwent laparoscopic segmentectomy for segments 2 and 8. Pathologic examination revealed two necrotic nodules located in the subcapsular areas of the liver. The lesions were slightly lobulated, and a small cystic change was observed in the nodule of segment 2, correlating with the MR imaging findings (Figure 4A). Microscopically, the insides of the lesions were composed of necrotic cells, and the edges of the lesions were rimmed by fibrous tissue (Figure 4B). The central zone of the nodules showed coagulative necrosis, and there was no evidence of malignancy. Inside the cystic space, acellular eosinophilic material was observed, but there was no parasite larva. No fungal organisms were identified on Gomori methenamine silver or Periodic Acid-Schiff stain, and no acid-fast bacilli were identified on Ziehl-Neelsen stain. Based on these findings, both lesions were diagnosed as solitary necrotic nodules of the liver.

## Discussion

We herein report a case of two incidentally found solitary necrotic nodules of the liver that mimicked hepatic metastases on imaging. Even with remarkable improvements in diagnostic imaging of the liver with CT and MR, some hepatic lesions are challenging to diagnose preoperatively. Solitary necrotic nodules are benign lesions, with no reported cases of serious complications, such as malignant transformation. Thus, to avoid unnecessary surgical exploration, radiologists should be aware of the imaging findings associated with solitary necrotic nodules of the liver.

Solitary necrotic nodules of the liver are mostly small, well-defined lesions measuring less than 3.0 cm with a round, oval, or lobulated shape [4,5]. They are commonly located in the right lobe and in the subcapsular areas of the liver [4,5]. Although most lesions are solitary, there have been reports of multiple lesions in a single patient [5], such as in the present case.

On contrast-enhanced CT, solitary necrotic nodules usually present as well-defined low- or iso-attenuated nodules. Usually, the lesions show no contrast-enhancement, but thin rim-enhancement may be seen [6]. The lesion may also have calcifications [7]. These findings overlap with those of hepatic metastases, which commonly appear as low-attenuated lesions and occasionally with rim-enhancement [8,9], making it very difficult to differentiate solitary necrotic nodules from hepatic metastases on CT alone.

Geng et al. [4] reported that solitary necrotic nodules may show low- or iso-signal intensity on T1WI and variable signal intensity on T2WI on MR imaging depending on degree of necrotic change. Intra-lesional T2 bright signal intensity may be seen due to cystic change within necrosis [4], such as in the present case. On contrast-enhanced MR imaging, Geng et al. [4] noted that none of the lesions showed contrast enhancement on all phases. However, other studies have reported that thin rim-enhancement may be seen due to the fibrous capsule [6], such as in our case.

PET/CT is a clinically useful imaging modality not only for staging or follow-up of malignancies, but also for further evaluation of suspicious findings on CT or MR imaging [10]. However, in cases of solitary necrotic nodules, variable FDG uptake has been reported depending on extent of necrosis [11]. Thus, FDG PET/CT may have limited value in differentiating solitary necrotic nodules from hepatic metastases.

## Conclusion

In conclusion, solitary necrotic nodule of the liver is a benign entity that usually shows low attenuation on contrast-enhanced CT and variable signal intensity on T2WI of MR imaging. Absence of contrast enhancement on CT or MR imaging may be helpful in differentiation of solitary necrotic nodules from hepatic metastases. However, thin rim-enhancement may also be seen due to the fibrous capsule. Radiologists should keep solitary necrotic nodules in mind as a possible differential diagnosis when hypovascular hepatic nodules are relatively small, solitary, or located in the subcapsular areas of the liver.
